# D-Pinitol Content and Antioxidant and Antidiabetic Activities of Five *Bougainvillea spectabilis* Willd. Cultivars

**DOI:** 10.3390/ph16071008

**Published:** 2023-07-15

**Authors:** Fatma Abo-Elghiet, Amal H. Ahmed, Hanan F. Aly, Eman A. Younis, Mohamed A. Rabeh, Saad Ali Alshehri, Khalid S. A. Alshahrani, Shaza A. Mohamed

**Affiliations:** 1 Department of Pharmacognosy and Medicinal Plants, Faculty of Pharmacy (Girls), Al-Azhar University, Nasr City, Cairo 11754, Egypt; elmerigy@yahoo.com (A.H.A.); shaza.halim@hotmail.com (S.A.M.); 2 Department of Therapeutic Chemistry, National Research Centre (NRC), El Behouth St., Giza 12311, Egypt; hanan_abduallah@yahoo.com (H.F.A.); youniseman530@yahoo.com (E.A.Y.); 3 Department of Pharmacognosy, College of Pharmacy, King Khalid University, Abha 62521, Saudi Arabia; mrabeh@kku.edu.sa (M.A.R.); salshhri@kku.edu.sa (S.A.A.); 4 College of Pharmacy, King Khalid University, Abha 62521, Saudi Arabia; khalidshhrani@gmail.com

**Keywords:** *Bougainvillea spectabilis*, cultivars, D-pinitol, antidiabetic, antioxidant

## Abstract

Diabetes mellitus is a major challenge for global health, and *Bougainvillea spectabilis* Willd. (*B. spectabilis*) is a widely used herbal remedy with diverse cultivars traditionally used for diabetes treatment. However, the comparative efficacy of these cultivars remains ambiguous. This study aimed to evaluate the D-pinitol content and DPPH radical-scavenging activity of methanolic leaves extracts of five *B. spectabilis* cultivars. Furthermore, the effects of these cultivars on various parameters, including blood glucose levels, oxidative stress markers, inflammatory cytokines, lipid profiles, liver enzymes, renal function markers, and histopathological changes, were assessed in STZ-induced diabetic rats after one month of oral daily treatment. All tested cultivars demonstrated significant improvements in the measured parameters, albeit to varying extents. Notably, the LOE cultivar, distinguished by its orange bracts, exhibited the highest efficacy, surpassing the effectiveness of glibenclamide, an antidiabetic medication, and displayed the highest concentration of D-pinitol. These findings underscore the importance of carefully selecting the appropriate *B. spectabilis* cultivar to maximize the antidiabetic efficacy, with a particular emphasis on the correlation between antidiabetic activity and D-pinitol concentrations.

## 1. Introduction

As stated by the International Diabetes Federation (IDF), diabetes mellitus (DM) is a metabolic disorder that disrupts the body’s ability to regulate blood sugar levels and has reached the status of a global epidemic. According to IDF’s report in 2021, the number of affected adults with DM was 537 million, and projections indicate a rise to 643 million by 2030 and 783 million by 2045 [1]. DM can result from either insufficient insulin production (type 1DM) or a deficiency in insulin action due to obesity and lifestyle changes (type 2DM). Regrettably, diabetes is a leading cause of various complications, such as nonalcoholic fatty liver disease, hyperlipidemia, kidney failure, cardiovascular diseases, blindness, and lower limb amputation [2]. At present, herbal medicine is gaining popularity as a means of managing diabetes due to the adverse effects associated with oral hypoglycemic drugs and their inability to cure the disease completely [3,4].

*Bougainvillea* is a genus in the Nyctaginaceae family that includes approximately 18 species with numerous cultivars, distinguished primarily by the color of their bracts [5]. Other features, such as bract and leaf size, foliage diversification, star, floral tube, stamen positions, pubescence, and flowering behavior, also contribute to the cultivar variation [6]. One well-known species in this genus is *Bougainvillea spectabilis* (*B. spectabilis*), commonly known as Great Bougainvillea or Bougainvillea, which is native to South America and extensively cultivated in tropical and subtropical areas for its ornamental value, attributed to its colorful bracts [7,8]. Different cultivars of *B. spectabilis* have emerged through natural or induced mutations [9,10], exhibiting a diverse range of bract colors, including pink, purple, red, yellow, orange, magenta, white, and bi-colored varieties [11,12]. These cultivars have been extensively studied and are highly valued for their beauty and attractiveness in landscaping and horticulture.

Apart from its ornamental beauty, *B. spectabilis* has traditionally been used in various countries to treat a range of health problems. For example, in Mexico, it has been used to treat respiratory and cough problems, Leucorrhea, and Parkinson’s disease, while in Tamil Nadu’s Kolli hills, it is considered a traditional remedy for inflammation. *B. spectabilis* leaves are also used to reduce stomach acidity and ulcers [13]. In Indian traditional medicine, *B. spectabilis* leaves have been utilized to manage diabetes [8,14,15] and are included in different herbal formulations for diabetes treatment [16,17,18,19]. The plant’s traditional uses have been supported by numerous studies, particularly its hypoglycemic effect [20,21,22,23].

*B. spectabilis* contains various phytoconstituents, including flavonoids, phenolics, alkaloids, tannins, volatile oil, saponins, terpenoids, steroids, and D-pinitol [24]. D-pinitol, also known as 3-*O*-methyl-chiro-inositol, is a prominent phytoconstituent found in significant amounts within the leaves of *B. spectabilis* and considered the key contributor to the plant’s antidiabetic properties. It exhibits insulin-like properties and protects pancreatic tissue from oxidative stress caused by free radicals, while also possessing hepatoprotective, antihyperlipidemic, and anti-inflammatory activities [25].

The pharmacological properties of different *B. spectabilis* cultivars may vary due to differences in their phytochemical compositions or concentrations. For instance, a study found that the methanolic flower extracts of five *B. spectabilis* cultivars with various bract colors exhibited varying antibacterial and cytotoxic activities [26]. However, there is a lack of comparative research on the reported pharmacological effects of these different cultivars, despite the extensive use of the plant in folk medicine. Thus, it is crucial to compare the efficacy of different *B. spectabilis* cultivars to identify the one that is most effective as a natural remedy. In this context, the aim of this work was to compare the in vivo antidiabetic effects, antioxidant properties, and concentrations of D-pinitol, known for its antidiabetic efficacy, among methanolic leaves extracts of five *B. spectabilis* cultivars with different bract colors (red, white, orange, pink, and magenta) to determine their relative antidiabetic potency. The cultivars were selected based on their availability in Egypt, where the study samples were obtained.

## 2. Results

### 2.1. Yields of Extracts

The yields of the methanolic extracts of each *B. spectabilis* cultivar were determined to be 14% for LRE (extract from leaves of the cultivar with red bracts), 18% for LWE (extract from leaves of the cultivar with white bracts), 14.5% for LOE (extract from leaves of the cultivar with orange bracts), 15.5% for LPE (extract from leaves of the cultivar with pink bracts), and 17% for LME (extract from leaves of the cultivar with magenta bracts).

### 2.2. D-Pinitol Content

**Figure 1:**
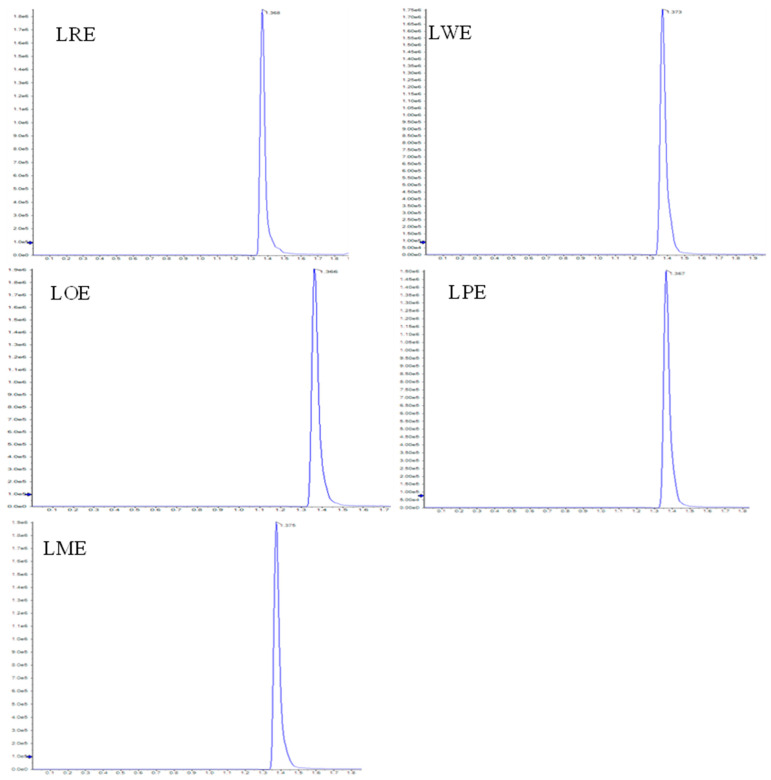
LC-ESI-MS/MS MRM chromatograms of the five tested *B. spectabilis* cultivars.

Using LC-ESI-MS/MS analysis, the D-pinitol content was quantified in the leaves methanolic extracts of the five *B. spectabilis* cultivars. The concentrations of D-pinitol, as indicated in Table 1, ranged from 5.08 to 6.95 mg/g extract. Among the cultivars, the LOE cultivar had the highest concentration of D-pinitol, while the LRE cultivar displayed the lowest concentration. Figure 1 presents LC-ESI-MS/MS MRM chromatograms of leaves methanolic extracts of the five tested *B. spectabilis* cultivars, demonstrating the targeted detection and quantification of D-pinitol using the LC-ESI-MS/MS technique in the multiple-reaction monitoring (MRM) mode.

### 2.3. DPPH^•^ Scavenging Activity

**Figure 2:**
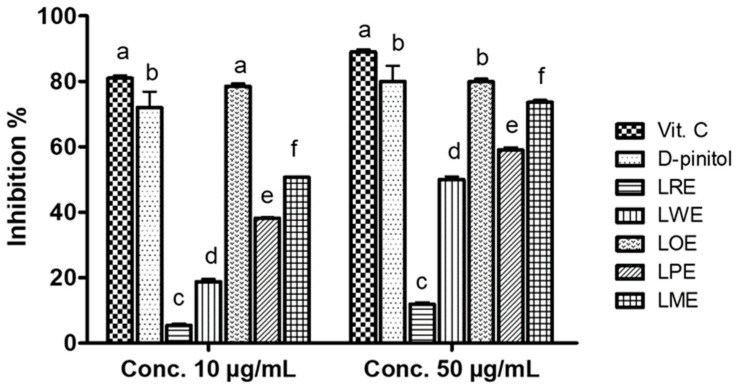
DPPH^•^ scavenging activity of D-pinitol and methanolic leaves extracts of the five tested *B. spectabilis* cultivars compared to vitamin C as reference standard. Data are means ± SD of three replicates in each group. The mean values within the bars that share the same superscript letters indicate no significant differences, while those with different superscript letters indicate significant differences (*p* < 0.05).

The in vitro antioxidant properties of *B. spectabilis* cultivars and D-pinitol were evaluated using the DPPH^•^ scavenging assay and compared to vitamin C as a reference standard. Figure 2 shows that, at a concentration of 10 µg/mL, the LOE cultivar exhibited the highest free-radical-scavenging activity (78.47%), which was significantly higher than that of D-pinitol (72%) and nearly equivalent to vitamin C (81%). When the concentration was increased to 50 µg/mL, both the LOE cultivar and D-pinitol displayed maximum inhibitory percentages (79.9% and 80%, respectively) compared to vitamin C (89%).

### 2.4. Antidiabetic Activity

#### 2.4.1. Acute Toxicity Study

Throughout the experimental period, no behavioral changes, mortality rates, or indications of toxicity were observed. The LD_50_ of the extracts was estimated to be greater than 4 g/kg b.wt. Based on these results and previous antidiabetic studies conducted on *B. spectabilis* [22], a dosage of 400 mg/kg b.wt. was selected for subsequent studies.

#### 2.4.2. Fasting Blood Glucose Levels

**Figure 3:**
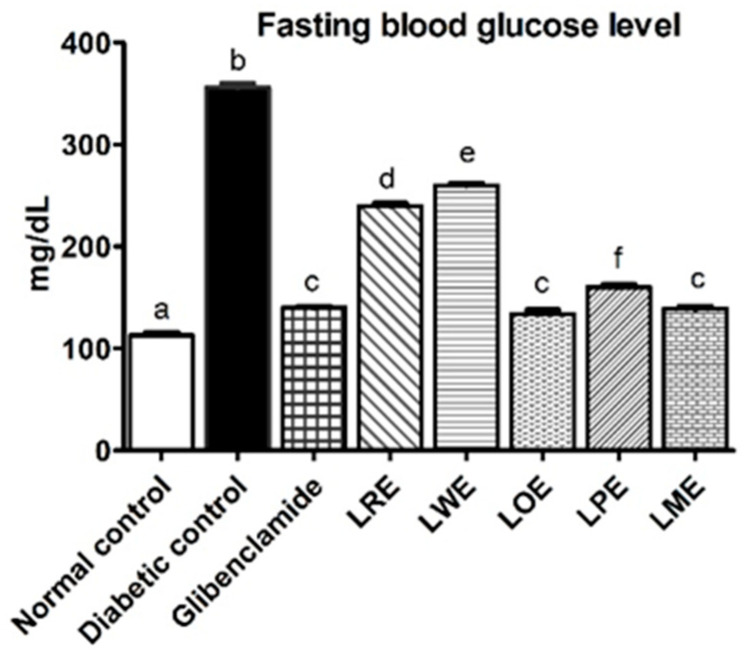
Effects of methanolic leaves extracts of various *B. spectabilis* cultivars and the standard drug glibenclamide on fasting blood glucose level in STZ-induced diabetic rats. Data are presented as mean ± SD (n = 7). The mean values within the bars that share the same superscript letters indicate no significant differences, while those with different superscript letters indicate significant differences (*p* < 0.05).

Streptozotocin (STZ) diabetic rats displayed significantly higher blood glucose levels (*p* < 0.05) compared to the normal control rats, showing a percentage change of 215%. Notably, oral treatment with LOE and LME cultivars demonstrated the most promising effects in reducing blood glucose levels, with no significant difference from the effect of the glibenclamide standard, as shown in Figure 3. These effects were followed by those of LPE, LRE, and LWE cultivars in decreasing order.

#### 2.4.3. Oxidative Stress Markers

**Figure 4:**
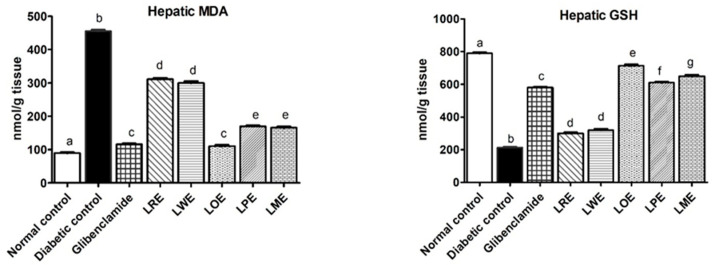
Effects of methanolic leaves extracts of various *B. spectabilis* cultivars and the standard drug glibenclamide on hepatic MDA and hepatic GSH levels in STZ-induced diabetic rats. Data are presented as mean ± SD (n = 7). The mean values within the bars that share the same superscript letters indicate no significant differences, while those with different superscript letters indicate significant differences (*p* < 0.05).

Figure 4 illustrates that the induction of diabetes led to a significant decrease (*p* < 0.05) in hepatic glutathione (GSH) levels (213 nmol/g tissue) and an increase in hepatic malondialdehyde (MDA) levels (455 nmol/g tissue) compared to the normal control group (790 and 89.88 nmol/g tissue, respectively). Following one month of oral treatment with different *B. spectabilis* cultivars, the hepatic MDA levels decreased and the hepatic GSH levels increased in STZ-induced diabetic rats, with the extent of improvement varying depending on the cultivar. Particularly, the LOE cultivar demonstrated the most significant improvements, surpassing the effects of the standard drug glibenclamide. The LME and LPE cultivars also exhibited considerable effects, while the LRE and LWE cultivars had the least impact.

#### 2.4.4. Pro-Inflammatory Markers and Adhesion Molecules

**Figure 5:**
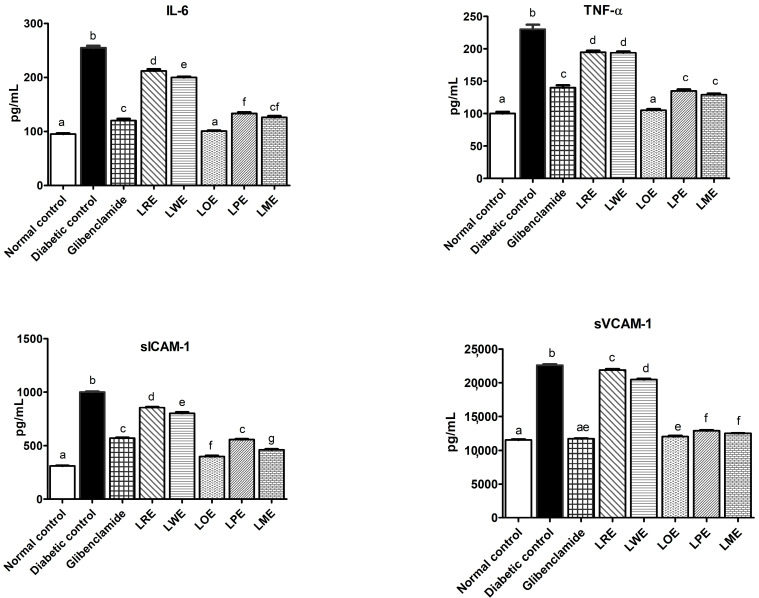
Effects of methanolic leaves extracts of various *B. spectabilis* cultivars and the standard drug glibenclamide on serum levels of IL-6, TNF-α, sVCAM-1, and sICAM-1 in STZ-induced diabetic rats. Data are presented as mean ± SD (n = 7). The mean values within the bars that share the same superscript letters indicate no significant differences, while those with different superscript letters indicate significant differences (*p* < 0.05).

Figure 5 demonstrates that diabetic rats displayed significantly elevated serum levels of interleukin-6 (IL-6), tumor necrosis factor-alpha (TNF-α), soluble vascular cell adhesion molecule-1 (sVCAM-1), and soluble intercellular adhesion molecule-1 (sICAM-1) compared to normal control rats. Upon oral treatment using methanolic leaves extracts of different *B. spectabilis* cultivars, varying degrees of improvement were observed in the levels of adhesion molecules and pro-inflammatory markers in STZ-induced diabetic rats. Among the tested cultivars, the LOE cultivar exhibited the highest efficacy in attenuating pro-inflammatory markers and adhesion molecules, outperforming the standard drug glibenclamide. Additionally, both LME and LPE cultivars demonstrated similar improvements to glibenclamide in TNF-α and sICAM-1 levels.

#### 2.4.5. Lipid Profiles

**Figure 6:**
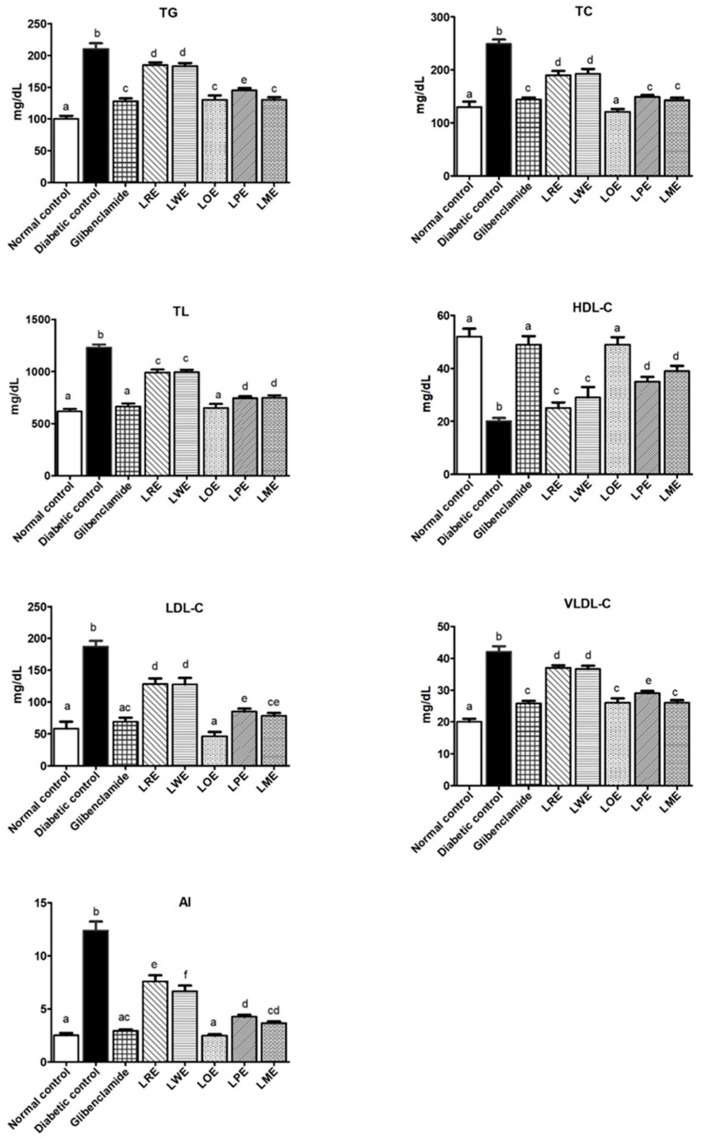
Effects of methanolic leaves extracts of various *B. spectabilis* cultivars and the standard drug glibenclamide on serum levels of TG, TC, TL, HDL-C, LDL-C, VLDL-C, and AI in STZ-induced diabetic rats. Data are presented as mean ± SD (n = 7). The mean values within the bars that share the same superscript letters indicate no significant differences, while those with different superscript letters indicate significant differences (*p* < 0.05).

The lipid profiles of diabetic rats exhibited significant changes (*p* < 0.05) in comparison to the normal control group. These changes were characterized by a notable increase in total lipid (TL), triglycerides (TG), total cholesterol (TC), very low-density lipoprotein cholesterol (VLDL-C), low-density lipoprotein cholesterol (LDL-C), and atherogenic index (AI) levels, accompanied by a significant decrease in high-density lipoprotein cholesterol (HDL-C) levels. However, treatment of these diabetic rats with methanolic leaves extracts of various *B. spectabilis* cultivars resulted in a significant improvement in their lipid profiles, with the extent of improvement varying depending on the specific cultivar used. Both the LOE and LME cultivars demonstrated remarkable effects, showing improvements comparable to those achieved with the standard drug glibenclamide. Intriguingly, the LOE cultivar exhibited superior performance in reducing TC levels. These findings are illustrated in Figure 6.

#### 2.4.6. Renal Function Markers

**Figure 7:**
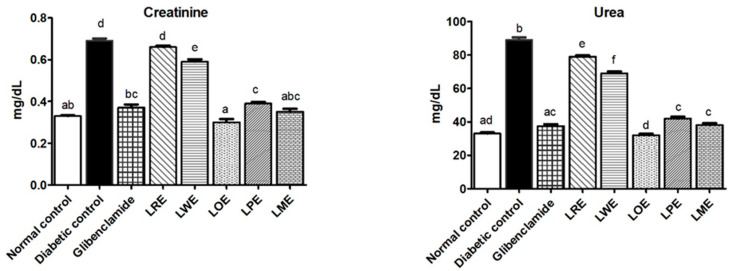
Effects of methanolic leaves extracts of various *B. spectabilis* cultivars and the standard drug glibenclamide on serum levels of creatinine and urea in STZ-induced diabetic rats. Data are presented as mean ± SD (n = 7). The mean values within the bars that share the same superscript letters indicate no significant differences, while those with different superscript letters indicate significant differences (*p* < 0.05).

The study evaluated the impact of methanolic leaves extracts of various *B. spectabilis* cultivars on the renal function of diabetic rats through monitoring their serum levels of creatinine and urea. Figure 7 illustrates the results obtained from the study. The STZ-diabetic group displayed significantly elevated levels of urea and creatinine (89 and 0.69 mg/dL, respectively) in contrast to the normal control group (33 and 0.33 mg/dL, respectively). However, oral administration of extracts from different *B. spectabilis* cultivars improved the renal function, as evidenced by reduced serum levels of creatinine (ranging from 0.66 to 0.30 mg/dL) and urea (ranging from 79 to 32 mg/dL). Notably, among the cultivars, LOE demonstrated the most pronounced effects in lowering both creatinine and urea levels. Interestingly, the effects of LOE were found to be more significant (*p* < 0.05) than those of the standard drug glibenclamide.

#### 2.4.7. Liver Function Parameters

**Figure 8:**
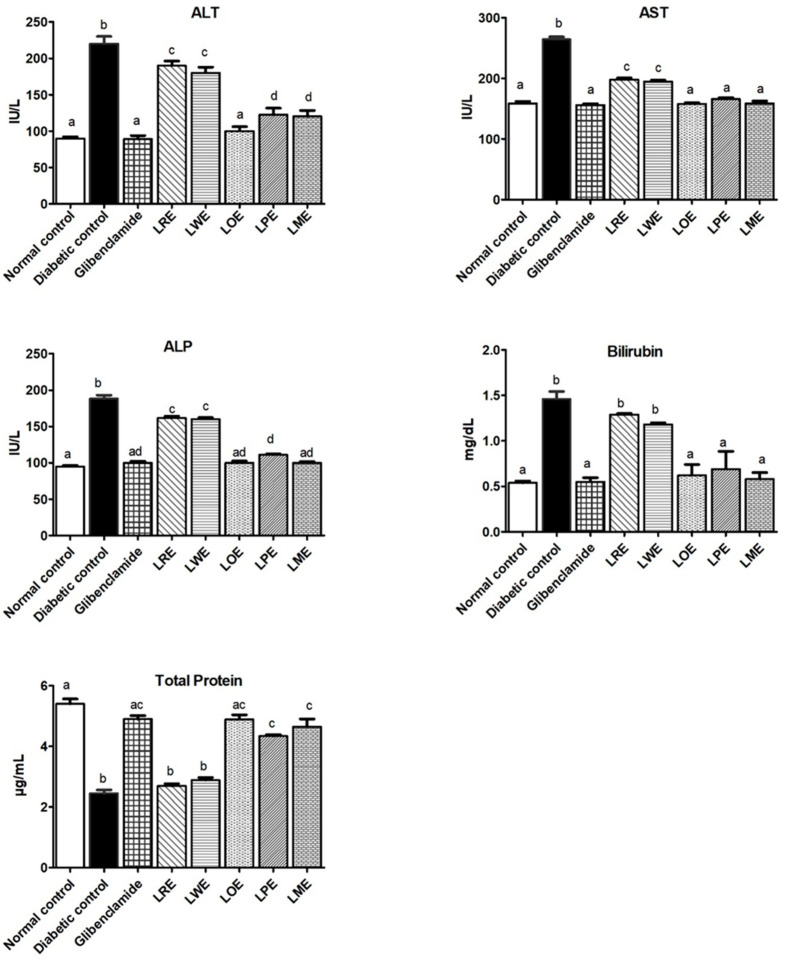
Effects of methanolic leaves extracts of various *B. spectabilis* cultivars and the standard drug glibenclamide on serum levels of ALT, AST, ALP, bilirubin, and total protein in STZ-induced diabetic rats. Data are presented as mean ± SD (n = 7). The mean values within the bars that share the same superscript letters indicate no significant differences, while those with different superscript letters indicate significant differences (*p* < 0.05).

Figure 8 demonstrates a notable increase in ALP, AST, ALT, and total bilirubin levels in the STZ-diabetic group in comparison to the control group (*p* < 0.05), along with a decrease in total protein levels. However, the daily oral administration of 400 mg/kg methanolic leaves extracts of various *B. spectabilis* cultivars effectively improved liver function. Particularly, the LOE, LME, and LPE cultivars displayed significantly higher efficacy in mitigating these alterations when compared to the standard glibenclamide treatment.

#### 2.4.8. Histopathology of Liver and Pancreas

**Figure 9:**
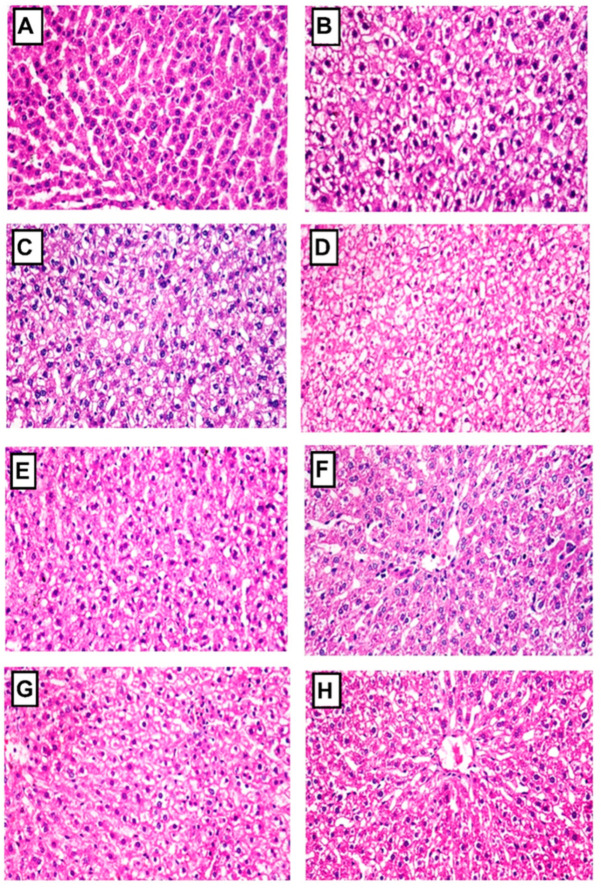
Photomicrographs of liver sections stained with hematoxylin and eosin, magnified at x400, from different experimental groups. (**A**) Normal control group: The photomicrograph shows a liver with a normal histological structure. (**B**) Diabetic group: The photomicrograph reveals severe vacuolar degeneration of hepatocytes. (**C**,**D**) Diabetic groups treated with LRE and LWE, respectively: The photomicrographs display moderate vacuolar degeneration of hepatocytes. (**E**–**H**) Diabetic groups treated with LOE, LPE, LME, and glibenclamide, respectively: The photomicrographs demonstrate mild vacuolar degeneration of hepatocytes.

The liver cells of the normal control rats exhibited a histological appearance characterized by a normal structure of the central vein and hepatocytes, as depicted in Figure 9. In contrast, STZ-induced diabetic rats displayed severe vacuolar degeneration in most hepatocytes. However, diabetic rats treated with *B. spectabilis* cultivars showed only mild vacuolar degeneration in a few hepatocytes compared to the standard drug, as shown in Figure 9. Additionally, Table 2 presents the scores for histopathological alterations observed in the livers of all treated groups.

**Figure 10:**
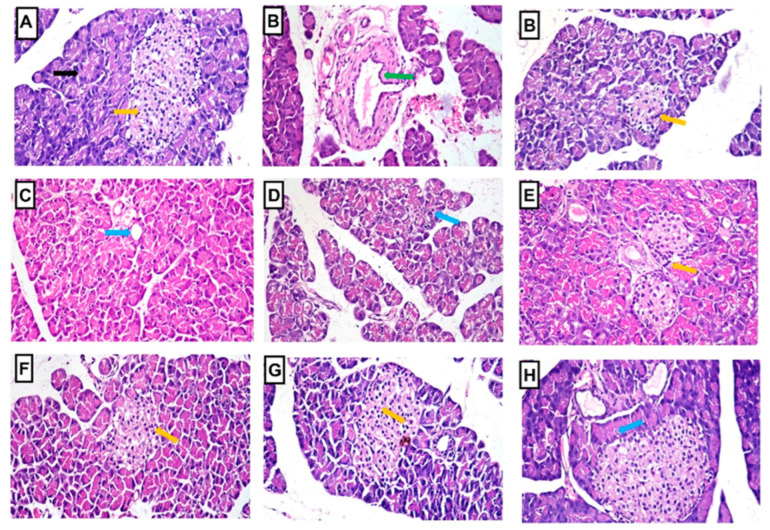
Photomicrographs of pancreatic sections stained with hematoxylin and eosin, magnified at x400, from various experimental groups. (**A**) Normal control group: The photomicrograph exhibits normal-sized islets of Langerhans (indicated by the orange arrow) and a normal exocrine pancreas (indicated by the black arrow). (**B**) Diabetic group: The photomicrograph reveals hemorrhage and periductal fibrosis (indicated by the green arrow), along with distortion and atrophy of the islets of Langerhans (indicated by the orange arrow). (**C**) Diabetic group treated with LRE: The photomicrograph displays moderate vacuolation and necrosis of the exocrine pancreas (indicated by the blue arrow). (**D**) Diabetic group treated with LWE: The photomicrograph shows mild vacuolar degeneration of some cells in the exocrine pancreas (indicated by the blue arrow). (**E**) Diabetic group treated with LOE: The photomicrograph indicates a mild restoration of the size of the islets of Langerhans (indicated by the orange arrow). (**F**) Diabetic group treated with LPE: The photomicrograph demonstrates a mild restoration of the size of the islets of Langerhans (indicated by the orange arrow). (**G**) Diabetic group treated with LME: The photomicrograph exhibits nearly normal-sized islets of Langerhans (indicated by the orange arrow) with a normal surrounding exocrine pancreas. (**H**) Diabetic group treated with glibenclamide: The photomicrograph shows mild vacuolar degeneration of a few cells in the exocrine pancreas (indicated by the blue arrow).

In comparison to the normal control rats, as illustrated in Figure 10, STZ-induced diabetic rats displayed various histopathological abnormalities in the pancreas, including distorted and atrophied islets of Langerhans, interstitial congestion, edema, hemorrhage, periductal fibrosis, and vacuolar degeneration and necrosis of the exocrine pancreas. However, the administration of *B. spectabilis* cultivars and the standard drug glibenclamide to STZ-induced diabetic rats resulted in a significant improvement in cellular damage, leading to the restoration of normal-sized islets of Langerhans and exocrine pancreatic tissue. The histopathological alteration scores observed in the pancreas of all treated groups are presented in Table 2.

## 3. Discussion

DM is a complex metabolic disorder characterized by high blood glucose levels due to insulin dysfunction, leading to various complications. This study evaluated the effect of methanolic leaves extracts of five different *B. spectabilis* cultivars on hyperglycemia and its associated complications in streptozotocin (STZ)-induced diabetic rats. The study also measured D-pinitol concentrations in the extracts. The findings emphasize the significant influence of cultivar selection on the effectiveness of *B. spectabilis* in managing diabetes and its associated complications.

In our study, we induced diabetes in rats by administering an intraperitoneal injection of STZ (50 mg/kg b.wt.). This injection caused damage to the pancreatic β-cells responsible for insulin secretion due to the generation of free radicals. This model effectively mimics aspects of human diabetes, including hyperglycemia, impaired insulin secretion, hyperlipidemia, and various types of organ damage [27,28]. Prior research [29,30] has indicated that D-pinitol, a bioactive compound found with a high content in *B. spectabilis* leaves, is known to increase plasma insulin levels, improve insulin sensitivity, normalize chronic hyperglycemia, and reduce glycosylated hemoglobin formation in STZ-induced diabetic rats. These mechanisms contribute to restoring glucose homeostasis and mitigating the harmful effects of chronic hyperglycemia. Furthermore, Devi and Ramesh [22] reported that the leaves of *B. spectabilis* contain flavonoids, which have the potential to enhance insulin secretion. Consistent with previous findings, our study showed that the daily oral administration of methanolic leaves extracts of different *B. spectabilis* cultivars (at a dose of 400 mg/kg body weight) for one month reduced fasting blood glucose levels in STZ-induced diabetic rats, although to varying degrees. Both LOE and LME cultivars showed similar improvements in lowering blood glucose levels comparable to the standard drug glibenclamide (at a dose of 10 mg/kg b.wt.). Conversely, the LPE, LRE, and LWE cultivars exhibited progressively lesser effects on blood glucose levels. Importantly, the histopathological examinations of the pancreas supported these biochemical results. These findings further support previous research suggesting that *B. spectabilis* extracts may act by enhancing insulin secretion or improving insulin sensitivity.

Oxidative stress, arising from an imbalance between reactive oxygen species (ROS) production and antioxidant defense systems, plays a crucial role in the pathogenesis and progression of diabetes and its complications. Chronic hyperglycemia in diabetes leads to increased ROS production, causing damage to cellular components such as proteins, lipids, and DNA and contributing to the development of complications including retinopathy, neuropathy, nephropathy, and cardiovascular diseases [31]. To evaluate the degree of oxidative stress, researchers measure lipid peroxidation levels and assess alterations in both non-enzymatic and enzymatic antioxidant activities within tissues. Glutathione (GSH), a crucial tripeptide molecule, acts as a primary non-enzymatic antioxidant, effectively neutralizing ROS, protecting cells from oxidative damage, and supporting other enzymes and cellular defense mechanisms [32]. Experimental studies have demonstrated that the polyunsaturated fatty acids in cell membranes are easily targeted by free radicals due to their multiple bonds. Lipid peroxidation, a critical biomarker for assessing ROS-induced oxidative stress, reflects the damage inflicted on lipids by free radicals, with malondialdehyde (MDA) levels commonly measured to estimate lipid peroxidation and evaluate oxidative stress [33].

Oxidative stress and hyperglycemia, which are prominent factors in diabetes, play a significant role in triggering inflammation by activating multiple signaling pathways, including NF-kB. This inflammatory response leads to the production of various pro-inflammatory molecules such as IL-6, TNF-α, sVCAM-1, and sICAM-1 [34]. These cytokines contribute to chronic low-grade inflammation, creating a detrimental cycle with insulin resistance. TNF-α promotes adipocyte lipolysis, affecting insulin-signaling and contributing to insulin resistance and glucose imbalance. Similarly, IL-6 also has an important role in insulin resistance [35]. Furthermore, IL-6 and TNF-α induce an inflammatory phenotype in vascular endothelial cells, causing morphological changes and increased expression of pro-inflammatory molecules, exacerbating inflammation within blood vessels [36]. Moreover, hyperglycemia and oxidative stress upregulate adhesion molecules on endothelial cells, facilitating immune cell recruitment and initiating atherosclerotic plaque formation. Additionally, the inflammatory environment impairs endothelial cell function, leading to endothelial dysfunction and related complications such as cardiovascular diseases [37,38].

Therefore, regulating the redox state represents a potential strategy to alleviate the oxidative stress and inflammation resulting from excessive ROS production in diabetes. In this work, STZ-induced diabetic rats exhibited significantly decreased hepatic levels of the antioxidant GSH and increased levels of the marker of lipid peroxidation, hepatic MDA, along with elevated serum pro-inflammatory cytokines (IL-6 and TNF-α) and serum endothelial adhesion molecules (sVCAM-1 and sICAM-1). However, treatment with methanolic leaves extracts of different *B. spectabilis* cultivars attenuated these changes to varying degrees. The LOE, LME, and LPE cultivars effectively restored hepatic GSH levels to close to normal, surpassing the effectiveness of the glibenclamide standard. The LOE cultivar exhibited similar effects to glibenclamide in reducing MDA levels, followed by the LME, LPE, LWE, and LRE cultivars, respectively. These findings align with previous studies that have reported the antioxidant activity of *B. spectabilis*, attributing this activity to bioactive constituents such as flavonoids and D-pinitol [29,30]. Our results also demonstrate strong DPPH^•^ scavenging activity of the LOE cultivar, similar to the standard vit. C. Furthermore, the oral treatment of diabetic rats with different *B. spectabilis* cultivars significantly reduced the levels of pro-inflammatory mediators (TNF-α and IL-6) and endothelial adhesion molecules (sVCAM-1, and sICAM-1). Notably, the LOE cultivar exhibited superior effects compared to the standard drug, followed by the LME, LPE, LWE, and LRE cultivars. The observed reduction in pro-inflammatory mediators is presumed to have potentially improved insulin-signaling pathways and the glucose uptake process. These findings highlight the potential of *B. spectabilis* cultivars as a natural source of antioxidants, capable of mitigating the detrimental effects of oxidative stress and inflammation induced by hyperglycemia, albeit with varying degrees of effectiveness. Therefore, the development of a drug possessing antioxidant, anti-inflammatory, and antidiabetic properties could prove beneficial in the management of diabetes.

Diabetic dyslipidemia is a common condition in individuals with diabetes that involves disruptions in the production and clearance of plasma lipoproteins. It is characterized by specific changes in lipid levels, including low levels of HDL-C, elevated levels of LDL-C (including small dense LDL-C), and increased levels of TG. These abnormalities significantly increase the risk of cardiovascular disease in people with diabetes. The development of diabetic dyslipidemia is influenced by various factors, with insulin resistance and hyperglycemia playing crucial roles. Insulin resistance leads to increased lipolysis and subsequent elevation of free fatty acid flux, a key contributor to the abnormal lipid profile observed in diabetes [39]. Other factors, such as the effects of insulin on apoprotein production, regulation of lipoprotein lipase, actions of cholesteryl ester transfer protein, and peripheral effects of insulin, also contribute to this condition [40]. Hyperlipidemia, characterized by elevated lipid levels, further exacerbates the condition by promoting free radical production, oxidative stress, and LDL-C oxidation, all of which are significant factors in the development of atherosclerosis. Atherosclerosis, characterized by plaque formation in the arteries, narrows blood vessels and increases the risk of cardiovascular events like heart attacks and strokes [41]. Recent research has highlighted the importance of calculating the atherogenic index (AI), as it provides valuable insights into the balance between protective and atherogenic lipoproteins. It has emerged as an important tool for predicting atherosclerosis, coronary heart disease, and the effectiveness of lipid-lowering therapies. In fact, AI has been found to be a more reliable predictor compared to evaluating LDL-C or HDL-C levels alone [42].

In our experimental model of STZ-induced diabetes in rats, we observed an abnormal lipid profile characterized by significantly increased plasma levels of TC, TL, TG, VLDL-C, LDL-C, and AI, along with decreased levels of HDL-C. These findings matched prior studies reported by Chauhan et al. and Madhuri and Naik [29,43]. Oral treatment using methanolic leaves extracts of various *B. spectabilis* cultivars significantly improved the abnormal lipid profile of these diabetic rats but to varying degrees. Among the cultivars tested, the LOE cultivar exhibited the most significant effects on all lipid profile parameters, which were better than or similar to the effects of glibenclamide, an antidiabetic medication. Other cultivars such as LME and LPE also showed beneficial effects, although to a lesser extent. The LRE and LWE cultivars had the least impact. These findings not only support previous research by Chauhan et al. and Saikia and Lama, which confirms the beneficial anti-hyperlipidemic effects of *B. spectabilis* leaves [29,44], but also emphasize the importance of selecting the appropriate cultivar for optimal outcomes. Additionally, previous studies have indicated the positive impact of D-pinitol, a bioactive component present in *B. spectabilis* leaves, in reducing diabetic hyperlipidemia [45]. Therefore, it is possible that the antihyperlipidemic effect of *B. spectabilis* leaves can be attributed, at least partially, to their D-pinitol content.

In the present study, we aimed to investigate the impact of diabetes on renal function and explore the potential benefits of various *B. spectabilis* cultivars in managing diabetic nephropathy, a prevalent complication of DM. The development of diabetic nephropathy is influenced by hyperglycemia, which can trigger various mechanisms such as increased oxidative stress, advanced glycation end products production, and activation of renin-angiotensin system and protein kinase C pathways. These mechanisms can lead to vascular inflammation and altered gene expression of growth factors and cytokines. A clinical hallmark of diabetic nephropathy is the decline in glomerular filtration rate, which is characterized by elevated levels of serum urea and creatinine [46]. STZ-induced diabetic rats exhibited significantly higher levels of serum creatinine and urea compared to the normal control group, indicating renal dysfunction, consistent with previous research [47]. Treatment of diabetic rats with methanolic leaves extracts of different *B. spectabilis* cultivars resulted in a reduction in serum creatinine and urea levels. Notably, the LOE cultivar showed superior efficacy in normalizing these biomarkers compared to the glibenclamide standard, while both LPE and LME cultivars exhibited similar improvements. These findings emphasize the importance of selecting the appropriate *B. spectabilis* cultivar for effective diabetes management.

Similarly, the liver is affected by similar underlying mechanisms in diabetes, including oxidative stress, chronic hyperglycemia, and metabolic abnormalities, which can lead to hepatic damage [48]. In our study, we observed a significant increase in serum liver enzymes (AST, ALT, and ALP) and bilirubin levels, along with a substantial decrease in total protein levels in diabetic rats. These findings are in line with previous research that evaluated liver damage associated with insulin insufficiency syndrome using similar analyses [49]. Elevated serum levels of ALP, AST, and ALT reflect liver cellular damage, as these enzymes are released from the liver cytosol into the blood stream. Conversely, low total serum protein levels indicate reduced production of albumin and other plasma proteins by the liver due to insulin insufficiency [48,49]. Elevated bilirubin levels can indicate both cholestasis and severe liver disease when hepatocyte function is significantly impaired [50]. The observed histopathological changes in the liver of diabetic rats further supported these liver function deteriorations. However, treatment with different *B. spectabilis* cultivars significantly improved liver function in the diabetic rats. This was evident through the reduction in the activities of ALP, AST, and ALT enzymes, as well as bilirubin levels. Moreover, the treatment increased total protein content and ameliorated the histopathological liver changes. The LOE cultivar exhibited comparable effectiveness to the standard glibenclamide in normalizing all investigated liver function biomarkers and improving histopathological findings. Both the LME and LPE cultivars showed similar effects to LOE and the standard glibenclamide in normalizing AST and bilirubin levels, with the LME cultivar showing additional benefits in normalizing ALP levels compared to the LPE cultivar. These findings underscore the potential of *B. spectabilis* cultivars for improving liver function in diabetes.

D-pinitol, a hypoglycemic phytoconstituent found in *B. spectabilis*, has demonstrated its ability to enhance glucose uptake and improve insulin sensitivity by activating the phosphatidylinositol 3-kinase/protein kinase B (PI3K/Akt) signaling pathway. This pathway facilitates the transportation of glucose from the bloodstream into the cells for utilization or storage. Furthermore, D-pinitol suppresses gluconeogenesis, leading to a decrease in blood glucose levels and an improvement in insulin sensitivity [51,52,53]. Given these properties of D-pinitol, it was important to determine its concentration in the tested *B. spectabilis* cultivars. To achieve this, we utilized the precise and sensitive LC-ESI-MS/MS technique for quantification. The results indicate significant variations (*p* < 0.05) in the levels of D-pinitol among the five *B. spectabilis* cultivars. Notably, the LOE cultivar, which consistently demonstrated superior results surpassing or equal to the standard glibenclamide throughout the experiment, exhibited the highest concentration of D-pinitol (6.95 mg/g extract). Although the antidiabetic activity of *B. spectabilis* may arise from the synergistic effects of all phytoconstituents, our findings highlight the significant role of D-pinitol in this activity, as evidenced by the relationship between its concentration and the antidiabetic effect of the cultivar.

One limitation of this study is the incomplete analysis of the chemical profile of all *B. spectabilis* cultivars. Although the identification of D-pinitol as a potential bioactive compound responsible for antidiabetic effects is promising, further investigation is necessary to gain a more comprehensive understanding of the variations in antidiabetic properties among the cultivars. Such an in-depth exploration would provide valuable insights into the specific compounds and mechanisms underlying the observed effects, thereby validating the findings and potentially facilitating the use of the extract as a complementary or alternative therapy for diabetes management.

## 4. Materials and Methods

### 4.1. Chemicals

Acetonitrile, formic acid, and water for HPLC grade were purchased from Merck (Darmstadt, Germany). Analytical grade methanol was obtained from El-Nasr Chemicals Co. (Giza, Egypt), while other compounds such as vitamin C, D-Pinitol standard (95%), streptozotocin (STZ), glibenclamide, and 2,2-diphenyl-1-picrylhydrazyl (DPPH) were obtained from Sigma-Aldrich (St. Louis, MO, USA).

### 4.2. Plant Material

In October 2021, the fresh aerial parts of five *Bougainvillea spectabilis* Willd. cultivars were collected from the Giza Zoo in Giza, Egypt (GPS coordinates 30°02′ N and 31°21′ E), in accordance with institutional, national, and international norms. The cultivars were identified based on their morphological variations, particularly their distinct colored bracts (red, white, orange, pink, and magenta), by Dr. Trease Labeb, a senior plant taxonomy specialist at the Orman Botanical Garden in Giza, Egypt. The plant’s botanical name was confirmed using the World Flora Online database (http://www.worldfloraonline.org) (accessed on 15 December 2022). Voucher specimens of the cultivars (BsR21, BsW21, BsO21, BsP21, and BsM21) were deposited separately in the Herbarium of Pharmacognosy and Medicinal Plants Department at Faculty of Pharmacy (Girls), Al-Azhar University, Cairo, Egypt.

### 4.3. Extraction

The extraction method was carried out according to Mandal et al. [20]. Briefly, the leaves of each cultivar were air-dried and crushed. One hundred grams of the resulting powder was macerated in methanol at room temperature for 48 h, with repeated maceration until exhaustion. The extracts were then filtered using Whatman No. 1 filter paper and labeled as follows: LRE (extract from leaves of the cultivar with red bracts), LWE (extract from leaves of the cultivar with white bracts), LOE (extract from leaves of the cultivar with orange bracts), LPE (extract from leaves of the cultivar with pink bracts), and LME (extract from leaves of the cultivar with magenta bracts). The extracts were concentrated via vacuum evaporation using a Buchi^®^ R-210 Rotavapor^®^ Evaporator (manufactured in Switzerland) at 45 °C.

### 4.4. LC-ESI-MS/MS Study

To quantify D-pinitol, a known phytoconstituent with antidiabetic activity, a volume of 5 μL of each cultivar sample was injected into an LC-ESI-MS/MS system. The system used for separation was the ExionLC AC system, while detection was performed using the SCIEX Triple Quad 5500+ MS/MS system with electrospray ionization (ESI). The separation process involved the use of a ZORBAX Eclipse Plus C18 Column (4.6 × 100 mm, 3.5 µm), with two mobile phases: X, which consisted of 0.1% formic acid in water, and Y, which was acetonitrile (LC grade). The mobile phase program was set as follows: 10% Y from 0–0.2 min, 10–50% Y from 0.2–3 min, 50% Y from 3–4 min, 50–10% Y from 4–5 min, and then held at 10% Y for an additional minute, with a flow rate of 0.7 mL/min. For detection, multiple-reaction monitoring (MRM) analysis of D-pinitol (*m*/*z*: 195.085/109.03) was performed in the positive ionization mode. The following parameters were used: curtain gas at 25 psi, ion spray voltage at 5500, source temperature at 500 °C, ion source gas 1 and 2 at 55 psi, with a de-clustering potential of 40, collision energy at 23, and collision energy spread at 10. To quantify the amount of D-pinitol in the sample, a calibration curve was constructed using D-pinitol dilutions ranging from 0.1 to 1000 ng/mL. The peak area of the sample was compared to the calibration curve to determine the concentration of D-pinitol present.

### 4.5. In Vitro Antioxidant Activity (DPPH^•^ Scavenging Assay)

The DPPH^•^ scavenging activity of methanolic leaves extract of each *B. spectabilis* cultivar was determined following the method outlined by Abo-Elghiet et al. [54]. In brief, DPPH solution was added to each sample solution, and the resulting mixture was allowed to react at room temperature for 30 min. The decolorization of DPPH from deep purple to pale yellow was indicative of the antioxidant’s ability to scavenge free radicals. The absorbance of each sample was then measured at a wavelength of 515 nm, relative to a blank control. Vitamin C was used as the reference standard in this assay. To assess the scavenging capacity of each extract, the DPPH^•^ scavenging efficiency (Inhibition %) was calculated using the following formula:

DPPH^•^ scavenging efficiency (Inhibition %) = [(A_b_−A_s_)/A_b_)] × 100

where A_b_ represents the absorbance of the blank control (DPPH + methanol). A_s_ represents the absorbance in the presence of the cultivars’ extracts.

### 4.6. In Vivo Antidiabetic Activity

#### 4.6.1. Animals

The study protocol for this research was approved by the Ethical Review Committee of the Faculty of Pharmacy (Girls), Al-Azhar University, with reference number 372. The study followed the guidelines outlined in the “Guide for the Care and Use of Laboratory Animals” [55]. For the experimental procedures, adult male albino Wistar rats were utilized. These rats were obtained from the animal house of the National Research Centre in Dokki, Giza, Egypt. The rats had an initial weight of approximately 160 ± 10 g and underwent a one-week acclimatization period in the laboratory. During this period, the rats were housed in steel cages under standard room temperature conditions of 26 ± 2 °C. The rats were maintained on a 12 h light and 12 h dark cycle, providing them with a regular day–night rhythm. They were given unrestricted access to both food and water throughout the acclimatization and experimental periods.

#### 4.6.2. Acute Toxicity Study

To determine a safe dose range for subsequent experiments, an acute toxicity test was conducted on mice using serial concentrations of plant extracts (LRE, LWE, LOE, LPE, and LME). The study followed the protocol described by Ferdous et al. [21]. For each extract, five groups of five mice each were administered either saline (group I) or the extract at concentrations of 500, 1000, 2000, and 4000 mg/kg b.wt. (groups II–V). The mice were then monitored for a duration of 72 h to detect any abnormalities or mortality.

#### 4.6.3. Experimental Design

A total of fifty-six rats were randomly divided into eight groups, each consisting of seven rats, as follows:

Group 1 (Normal control): Rats received an intraperitoneal injection of 0.1 M citrate buffer with pH 4.5, along with standard chow.

Group 2 (STZ-diabetic control): Rats were induced with STZ (streptozotocin) to develop diabetes and received standard chow.

Groups 3–7 (STZ-diabetic rats + *B. spectabilis* cultivar extracts): Each diabetic rat group received a daily oral dose of 400 mg /kg b.wt. of one of the following extracts (LRE, LWE, LOE, LPE, or LME), dissolved in distilled water.

Group 8 (STZ-diabetic rats + glibenclamide): Diabetic rats received a daily oral dose of the reference drug glibenclamide at a dose of 10 mg/kg b.wt..

#### 4.6.4. Induction of Diabetes

To induce experimental type 2 diabetes in the rats, we followed the protocol outlined by Jaishree and Narsimha (2020) and Sivakumar et al. (2010) [30,56]. In brief, after an overnight fast, the rats received a single intraperitoneal injection of 50 mg/kg b.wt. of STZ, which was dissolved in a freshly prepared 0.1 M citrate buffer with a pH of 4.5. To prevent hypoglycemic shock, a 10% glucose solution was administered to the rats 6 h after STZ injection and provided overnight. Fasting blood glucose levels were measured 72 h after the STZ injection using an Accu-Chek Active glucometer (Roche Diagnostics, Penzberg, Germany) by collecting blood samples from the rats’ tail veins. Rats with fasting blood glucose levels exceeding 250 mg/dL were deemed diabetic and selected for subsequent experiments. Treatment for the diabetic rats began on the fourth day after the STZ injection and continued for a duration of one month.

#### 4.6.5. Sample Preparation

At the end of the treatment period, following an overnight fast, the rats were anesthetized with diethyl ether. Sublingual blood samples were collected and then centrifuged to obtain serum, which was stored at −80 °C for later analysis. Afterwards, the rats were euthanized by cervical decapitation, and their livers and pancreases were excised and weighed. Half of each liver was homogenized in a phosphate buffer, and the resulting supernatant was stored at −80 °C for measuring MDA and GSH levels. The remaining half of each liver and the pancreas were fixed in 10% formalin for histological examination.

#### 4.6.6. Biochemical Investigations

The effects of methanolic leaves extracts of different *B. spectabilis* cultivars on diabetic rats were assessed by analyzing various parameters, including blood glucose levels, oxidative stress, pro-inflammatory markers, lipid profiles, and liver and renal function.

##### Estimation of Blood Glucose

For the assessment of fasting blood glucose levels, an Accu-Chek Active glucometer (Roche Diagnostics, Penzberg, Germany) was used.

##### Estimation of Oxidative Stress Markers

The levels of hepatic MDA [57] and hepatic GSH [58] were estimated using kits purchased from Biodiagnostic (Giza, Egypt).

##### Estimation of Pro-Inflammatory Markers

Serum levels of inflammatory cytokines, including TNF-α [59] and IL-6 [60], as well as serum endothelial adhesion molecule sICAM-1 [61] were measured using Quantikine^®^ ELISA kits obtained from R&D Systems (Minneapolis, Minnesota,, USA). Additionally, the levels of sVCAM-1 [61] were measured using EIAab^®^ ELISA kit (Wuhan, Hubei, China).

##### Estimation of Lipid Profiles

To evaluate the lipid profiles, serum levels of TC, TL, HDL-C, and TG were measured. Colorimetric kits from Biodiagnostic, Cairo, Egypt, were utilized following the methodology described by Ben Khaled et al. [62]. The levels of LDL-C and VLDL-C were calculated using Friedewald’s formula: VLDL-C = TG/5 and LDL-C = TC–(HDL-C + VLDL-C) [63]. Additionally, the AI was calculated as the ratio of TC to HDL-C [64].

##### Estimation of Renal Function

To assess renal function, serum levels of creatinine and urea were determined using colorimetric kits obtained from Biodiagnostic, Cairo, Egypt [65].

##### Estimation of Liver Function

Liver function was assessed by measuring serum levels of alkaline phosphatase (ALP) [66], aspartate and alanine aminotransferase (AST and ALT) [67], total bilirubin [68], and total protein [69] using kits provided by Biodiagnostic, Cairo, Egypt.

#### 4.6.7. Histopathological Investigation

Liver and pancreas samples were preserved, embedded in paraffin beeswax tissue blocks, and sectioned into 4 μm thick sections. Routine examination was performed using a light electric microscope after staining the sections with hematoxylin and eosin stain [70].

The histopathological changes observed in the liver and pancreas were documented and assigned a grading score ranging from 0 to 3. A score of 0 indicated no change, while scores of 1, 2, and 3 indicated mild, moderate, and severe changes, respectively. To provide a quantitative assessment, the grading was expressed as a percentage. Changes affecting less than 30% of the tissue were considered mild, changes affecting between 30% and 50% were considered moderate, and changes affecting more than 50% were considered severe [71].

### 4.7. Statistical Analysis

The collected data were subjected to statistical analysis using one-way analysis of variance (ANOVA) to assess significant differences between the groups. Subsequently, Tukey’s post hoc analysis was conducted for multiple comparisons. The results were reported as mean ± standard deviation (SD), with statistical significance set at *p* < 0.05.

The data were analyzed using one-way analysis of variance (ANOVA), followed by Tukey’s post-hoc analysis for multiple comparisons to determine significant differences between groups. Results were reported as mean ± standard deviation (SD), and statistical significance was set at *p* < 0.05. Different letters were used to denote significant differences between groups. The statistical analyses were performed using SPSS software (version 8, SPSS for Windows 7, Chicago, IL, USA).

## 5. Conclusions

The present study assessed the effects of the methanolic leaves extracts of five *B. spectabilis* cultivars on various aspects of diabetes in STZ-induced diabetic rats. The results demonstrated significant reductions in hyperglycemia, oxidative stress, hyperlipidemia, inflammation, and markers of renal and hepatic damage. Among the tested cultivars, the LOE cultivar exhibited the highest efficacy, outperforming the standard drug glibenclamide in multiple parameters. The superior effectiveness of the LOE cultivar can be attributed to its high concentration of D-pinitol, a potent anti-diabetic compound. These findings underscore the influence of cultivar-specific variations in achieving therapeutic benefits and support the *B. spectabilis* LOE cultivar as a promising alternative or complementary treatment to glibenclamide for effectively managing hyperglycemia and its associated complications.

## Figures and Tables

**Table 1:** D-pinitol concentrations in leaves methanolic extracts of various *B. spectabilis* cultivars.

Leaves Methanolic Extracts of *B. spectabilis* Cultivars	D-Pinitol Conc. (mg/g Extract)
LRE	5.08
LWE	5.25
LOE	6.95
LPE	6.17
LME	6.66

**Table 2:** Scoring of histopathological alterations in liver and pancreas of different experimental groups.

Groups	Liver Lesions	Pancreas Lesions			
	Vacuolar Degeneration of Hepatocytes	Distortion and Atrophy of Islets of Langerhans	Interstitial Congestion, Edema, and Hemorrhage	Periductal Fibrosis	Degeneration of the Exocrine Pancreatic Tissue
Normal control	0	0	0	0	0
STZ-diabetic control	3	3	3	3	3
Glibenclamide	1	0	0	0	1
LRE	2	1	1	1	2
LWE	2	1	1	0	1
LOE	1	1	0	0	1
LPE	1	1	1	0	1
LME	1	1	0	0	1

The score system was designed as follows: score 0 = absence of lesion in all rats of the group (n = 7), score 1 = lesion < 30%, score 2 = lesion of 30–50%, score 3 = lesion > 50%.

## Data Availability

Data are available upon request from the authors.
